# Sensors for Digital Transformation in Smart Forestry

**DOI:** 10.3390/s24030798

**Published:** 2024-01-25

**Authors:** Florian Ehrlich-Sommer, Ferdinand Hoenigsberger, Christoph Gollob, Arne Nothdurft, Karl Stampfer, Andreas Holzinger

**Affiliations:** 1 Human-Centered AI Lab, Institute of Forest Engineering, Department of Forest and Soil Sciences, University of Natural Resources and Life Sciences Vienna, 1190 Wien, Austria; florian.sommer@boku.ac.at (F.E.-S.); ferdinand.hoenigsberger@boku.ac.at (F.H.); karl.stampfer@boku.ac.at (K.S.); 2 Institute of Forest Growth, Department of Forest and Soil Sciences, University of Natural Resources and Life Sciences Vienna, 1190 Wien, Austria; christoph.gollob@boku.ac.at (C.G.); arne.nothdurft@boku.ac.at (A.N.)

**Keywords:** sensors, artificial intelligence, data quality, human-in-the-loop, digital transformation, smart forestry

## Abstract

Smart forestry, an innovative approach leveraging artificial intelligence (AI), aims to enhance forest management while minimizing the environmental impact. The efficacy of AI in this domain is contingent upon the availability of extensive, high-quality data, underscoring the pivotal role of sensor-based data acquisition in the digital transformation of forestry. However, the complexity and challenging conditions of forest environments often impede data collection efforts. Achieving the full potential of smart forestry necessitates a comprehensive integration of sensor technologies throughout the process chain, ensuring the production of standardized, high-quality data essential for AI applications. This paper highlights the symbiotic relationship between human expertise and the digital transformation in forestry, particularly under challenging conditions. We emphasize the human-in-the-loop approach, which allows experts to directly influence data generation, enhancing adaptability and effectiveness in diverse scenarios. A critical aspect of this integration is the deployment of autonomous robotic systems in forests, functioning both as data collectors and processing hubs. These systems are instrumental in facilitating sensor integration and generating substantial volumes of quality data. We present our universal sensor platform, detailing our experiences and the critical importance of the initial phase in digital transformation—the generation of comprehensive, high-quality data. The selection of appropriate sensors is a key factor in this process, and our findings underscore its significance in advancing smart forestry.

## 1. Introduction

Artificial intelligence (AI) has reached an impressive level of practical maturity [1]. AI makes it possible to solve real-world data problems in virtually all areas of application domains relevant to supporting human life [2]. Digital Transformation in Smart forestry [3], which integrates advanced technologies like AI and sensors into forest management and conservation, is crucial for enhancing sustainable practices, biodiversity protection [4,5], and climate change mitigation [6,7], which is fundamental to saving our planet without ruining human jobs [8]. When advances in smart forestry are combined with advances in AI, unimagined new solutions open up. This can help with many global problems and contribute to important sustainability development goals because forests are important carbon sinks and their conservation efforts are vital for the vision of climate neutrality by 2050 [9]. The effectiveness of advanced computational algorithms in AI is fundamentally reliant on the availability of large enough amounts of high-quality data that accurately reflect real-world scenarios [10].

This article delves into the application of these technologies in smart forestry and related environmental research, with a particular focus on the pivotal role of sensors in data acquisition. Traditional methods in these scientific fields, which have historically relied on data generation spanning decades, are evolving [11]. The conventional practice of analyzing data in isolation is being supplanted by more integrated approaches, recognizing the complexity of discerning multidimensional relationships between variables. This integration not only enhances the capabilities of AI algorithms but also merges a domain-specific expertise with a broader interdisciplinary understanding. Central to this evolution is the pursuit of a Human-Centered AI (HCAI) approach, where human domain expertise and experience are integrated early in the process, synergizing the strengths of both humans and machines for effective human–AI interaction. The quality of data input into these algorithms is a critical factor in bridging these areas. This paper details methods of data collection using various sensors in forest environments and examines their significance in driving digital transformation, underscoring the necessity of generating high-quality data for successful outcomes in this field.

Initially, the paper presents a succinct overview of sensor technologies under consideration for forest applications and digital transformation. This is followed by a comprehensive table delineating the optimal applications and advantages of each sensor system. The table also explores their potential implementation in forestry.

The subsequent section delves into a detailed examination of each technology. This analysis not only highlights the potential achievements within the realm of forestry but also incorporates insights from other disciplines, thereby offering a multidisciplinary perspective.

The third segment of this paper is dedicated to the operational aspects of these sensors in forest environments. It covers the intricacies of deployment, maintenance, and data collection processes. This section includes the novel integration of robots as mobile options for data generation on a large scale within the forest environment. In this chapter, a standardized equipment carrier solution for a different robotic chassis will be presented.

The concluding chapter synthesizes all these elements, illustrating how they can be integrated to benefit the research community and what can be achieved in the future. This integration leverages artificial intelligence and a diverse array of high-quality data, underscoring the transformation potential of these technologies in scientific research.

## 2. Digital Transformation in Smart Forestry Needs Sensors

Digital transformation is a concept at the forefront of contemporary academic and industrial discourse and refers to the integration of all types of digital technologies into all areas of a business or organization, fundamentally changing how they operate and deliver value to organizations [12]. This is not merely about a simple adoption of digital tools, but rather a holistic rethinking of whole business processes, strategies, and practices to leverage the capabilities of digital innovations [13,14].

The importance of digital transformation cannot be overstated. It represents a paradigm shift in the way organizations conceptualize and execute their business models over time [15]. In an era where data are the new currency, digital transformation enables organizations to harness the power of big data analytics, artificial intelligence, and machine learning to gain insights that drive smarter, faster business decisions. This shift is not confined to the private sector; public institutions and non-profits are also recognizing the imperative to adapt to this digital era.

Additionally, digital transformation fosters innovation by creating an environment where new ideas can be tested and implemented quickly. This agility is crucial in a rapidly changing market landscape, where the ability to adapt and innovate is often a key determinant of success. This is especially true for industries with a built-in disadvantage when utilizing such technologies due to their operating environment, in this case, forestry.

Digital transformation is not just a technological upgrade, but a strategic and operational overhaul that positions organizations to thrive in an increasingly digital world. Its importance lies in its capacity to revolutionize the way organizations operate, engage with customers, and innovate, ensuring their relevance and competitiveness in the 21st century. The advancement of digital transformation is underpinned by the development of cyber-physical systems, which are bolstered by progress in AI and machine learning, the proliferation of big data, and enhanced computational capabilities. A key challenge lies in the realm of multimodal information fusion, which involves integrating diverse data sources and elucidating to human experts the rationale behind specific outcomes. However, because AI is often sensitive to minor variations, and disturbances can significantly impact their outputs, a human-in-the-loop can sometimes (of course not always) help here to bring in conceptual understanding and experience; therefore, we propagate human experts as a part of future networked AI systems [3]. The possibility of involving a human-in-the-loop also has another advantage: since humans can quickly familiarise themselves with unknown processes by understanding the hypothetical input conditions under which the outcome changes, this can identify potential shortcomings in data collection at a very early stage [16]. Our integrated sensor network framework for smart forestry with a robotic base that interacts with various sensors and the human-in-the-loop can be seen in the graphical abstract, and will be described later.

Digital transformation in smart forestry represents a significant shift towards using advanced technologies for the sustainable management and conservation of forest resources. Central to this transformation is the generation and effective utilization of data, necessitating a variety of sensors and input devices. These devices are ideally designed to transcend singular research objectives, adhering to standardization principles that allow their data to be integrated into broader databases. This approach not only serves specific research needs but also enriches the collective knowledge base of the forestry community. In the realm of operational autonomy, sophisticated sensor technology is indispensable. For instance, LiDAR (Light Detection and Ranging) is instrumental in mapping forest topography, assessing biomass, and monitoring forest health, providing high-resolution 3D data for the precise measurements of tree height, canopy structure, terrain features and helping in the digital transformation of cable yarding for sustainable timber harvesting [17]. Similarly, multispectral and hyperspectral imaging plays a crucial role in monitoring vegetation health, species identification, and detecting changes in forest cover, capturing data across various wavelengths to reveal insights into plant health and ecological parameters.

Thermal imaging sensors are pivotal in detecting forest fires, monitoring wildlife, and assessing tree health, identifying areas of heat stress in trees or early stages of forest fires. Soil moisture sensors contribute significantly to understanding the water content in forest soils, informing irrigation practices and drought management strategies. The use of drones and aerial sensors offer a versatile platform for rapid and extensive forest monitoring, providing data on tree density, health, and signs of pest infestations or disease outbreaks. Additionally, acoustic sensors are employed for wildlife monitoring and biodiversity assessments, detecting and recording sounds of different animal species to aid in conservation efforts [18].

The digital transformation in smart forestry, therefore, is not merely about the adoption of technology but about integrating these technologies into a cohesive system that enhances sustainable forest management. By harnessing the power of data and automation, forestry operations can become more efficient, environmentally friendly, and responsive to the dynamic needs of our global ecosystem—sensors play a central role in achieving this.

The subsequent Table 1 provides an overview of various sensors utilized in the context of forestry, each with some advantages and disadvantages. This selection is based on our own experiences, which, while extensive, are inherently subjective and not all-encompassing. It is intended as an initial reference point for those new to the field. In the following we supplement this table with background information and a summary of related work, offering a well-rounded introduction from our perspective.

Previous sections delineated the requisite sensor modalities, culminating in an exposition on the necessity for heterogeneous data within fully functional AI architectures. Central to modern AI paradigms is machine learning, predicated on the acquisition of high-fidelity data that approximates real-world phenomena as accurately as possible. Effective training demands high-quality data that closely resembles real-world conditions. Some sensor operation platforms with advantages and disadvantages can be seen in Table 2. A significant challenge for many algorithms is the reduced effectiveness when applied to real-world data, which is often due to the increased variability and sometimes poor data quality. To mitigate these challenges, incorporating a wide array of inputs is essential. This methodology facilitates the algorithms’ capacity to detect intricate patterns and establish correlations among variables, which might surpass human analytical capabilities, consequently associating them with distinct results. The incorporation of an extensive range of variables diminishes the propensity for spurious inferences attributable to deficient datasets. Consequently, the system is proficient in assimilating all inputs and judiciously attributing proportional significance to each.

To illustrate with a specific example from forestry, assessing forest road trafficability can employ various technologies. These include the falling weight deflectometer [19], soil moisture measurements [20], and integrating these methods with different terrain models [21]. While these technologies are established and effective for evaluating road trafficability, AI introduces an advanced approach. It integrates all available data from the area to generate predictions. This integration may encompass surface temperature, recent rainfall, traction data from vehicles that have recently traversed the road, and even camera imagery from the specific road. An AI system leverages this comprehensive dataset to predict road conditions. Its accuracy improves with the availability of complete data, but even with partial data, it holds a distinct advantage over traditional methods. For instance, lacking recent soil moisture data, the AI can infer the likely moisture level using other available information, thus still providing a reliable prediction. Here, the role of the human-in-the-loop becomes crucial in the AI system [22]. The expert’s knowledge is invaluable for validating the feasibility of AI predictions and, if necessary, supplementing information that the system might lack.

## 3. Sensor Deep Dive

In the following, we present a section on a variety of sensors and observational platforms. These include locator devices, climate and weather sensors, temperature, moisture and humidity sensors, and soil pH sensors. Additionally, we cover a range of imaging technologies such as RGB cameras, thermal imaging cameras, and LiDAR. The section also delves into spectrometers and multispectral cameras, and concludes with a discussion on observational platforms, including satellites and Unmanned Aerial Vehicles (UAVs).

### 3.1. Locator Devices

Exactly knowing a location and linking obtained data to a specific location is of major importance. This is the field where Global Navigation Satellite Systems (GNSS) come into play. Probably the best-known system is the Global Positioning System (GPS), i.e., NAVSTAR [23], followed by Galileo, GLONASS, and Beidou [24].

Localization is of key importance in forestry and other unstructured environments, as the signal transmittance can be obstructed massively—usually one can expect positional accuracy within approximately 5 m of a true position in open sky settings, 7 m in young forest conditions, and 10 m under closed canopies [25]; recent results show better accuracy—this is theoretical up to 1.5 m [26]; however, it is highly dependent on the density of the forest and often there is no signal at all. Therefore, GNSS is rarely used alone but linked to technologies like LiDAR (explained later) to obtain the best location in such unstructured environments [27].

**Figure 1:**
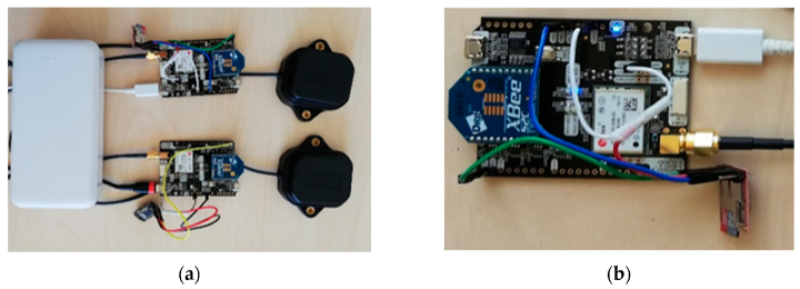
SimpleRTK2B boards and other used equipment: (**a**) SimpleRTK2B boards connected to ANN-MB-00 antennae and power bank for power supply; (**b**) Connection of SimpleRTK2B board with OpenLog DEV-13955 [30].

In general, low-cost GNSS receivers do not offer the same performance as high-quality receivers and antennas, but their performance is improving rapidly [28,29]. Cheap options will bring the benefit of adding locations to almost any measurement taken at a very low price point, improving overall data quality. Figure 1 shows a low-cost setup for positioning utilizing GNSS. We are aware that locator devices are mounted to most of today’s unmanned and manned vehicles; nevertheless, the importance of a precise location is key for the implementation of AI in forestry. Location is the one common denominator between all the different data types that can be collected; therefore, it is paramount to know which data come from the same location, especially when signal issues arise due to a dense canopy communication between known locations within the forest; maneuverable devices are important to achieve location-specific data.

Radio Frequency Identification (RFID) tags, which communicate with readers through radio waves, are extensively used for tracking objects or individuals, particularly in inventory management and asset tracking, or, e.g., in the forestry supply chain from tree felling to the sawmill and beyond [31]. Wi-Fi positioning systems determine a device’s location using the strength of nearby Wi-Fi signals and are commonly used in indoor navigation systems [32]; however, there are also some examples found outdoors, in precision agriculture [33] and forestry [34].

Bluetooth beacons, known as small radio transmitters, interact with nearby devices to offer location-based information and services, and are often found in retail environments and for indoor navigation purposes. Cellular network triangulation, which approximates the location of a mobile device by measuring the signal strength from multiple cell towers, provides a less accurate but useful positioning method in areas where GPS signals are weak or unavailable. When Bluetooth is used, a hierarchical approach is followed, i.e., mobile devices and sensors transmit their data over short distances, and smartphones and tablets serve as an intermediary data collection and processing centers for information that can then be transmitted via radio network systems or satellite communications. Data with greater spatial and temporal complexity is usually processed incrementally at lower levels and then merged and summarised at higher levels [35].

### 3.2. Climate and Weather Sensors

#### 3.2.1. Temperature Sensors

Temperature measurements of surfaces and the atmosphere are long-known concepts, and multiple cheap and simple setups exist. Temperature data are always closely linked to other climate parameters. As those types of sensors have existed for years, there are almost no options for pricing anymore, due to advances in cheap electronic components.

This has led to ideas for powering those sensors in remote environments for long periods of time. An example of this is the use of a Microbial Fuel Cell that powers a temperature sensor [36]. The advantage would be the ability to have the sensor out in the environment indefinitely, as long as biological material is available to fuel the Microbial Fuel Cell. Ideas like that showcase the potential for large amounts of data generated that can then be utilized by Artificial Intelligence to link it with other information.

One of the most prominent usecases for temperature measurements in forestry is the estimation of the temperature buffering capabilities of forests. Those buffering capabilities are of high interest due to the current global warming issues. Understanding microclimates created by forests is therefore of major importance and involves all kinds of temperature-measuring devices [37,38].

#### 3.2.2. Moisture and Humidity Sensors

Moisture and humidity are important factors to access in forestry. Ground moisture, for example, can provide an indication of road trafficability [39], where humidity provides insights in the microclimate below the tree coverage [40]. The important part of moisture and humidity sensors is the fact that they are becoming smaller, cheaper, and more robust. Small and cheap sensors are a great option to establish a sensor network permanently or semi-permanently. Cheap solutions can enable a simple automated exchange of sensors if failure is detected.

Hardie (2020) [41] carried out a review outlining a wide variety of novel sensor technologies that can be used in moisture monitoring. Most importantly, mobile sensor options were highlighted, for example, Near Infrared Sensors. With such technologies, it would be possible to cover larger areas and obtain for example a better understanding of moisture levels in a forest road throughout its route.

**Figure 2:**
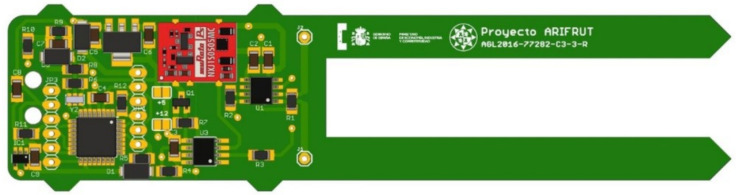
Experimental sensor based on a PCB [42].

Similar to temperature sensors, humidity sensors have found their way into cheap and small electronic components with the rise of Arduino and Raspberry Pi. A huge move has happened from different manufacturers to bring prices down and enhance the versatility of such systems. It is important to note that those sensors need to be checked regularly with standardized methods to ensure proper function. Nevertheless, such cheap options open up the possibility of large-scale data collection. Figure 2 showcases a simple PCB-based setup for a soil moisture sensor.

### 3.3. Soil pH Sensors

**Figure 3:**
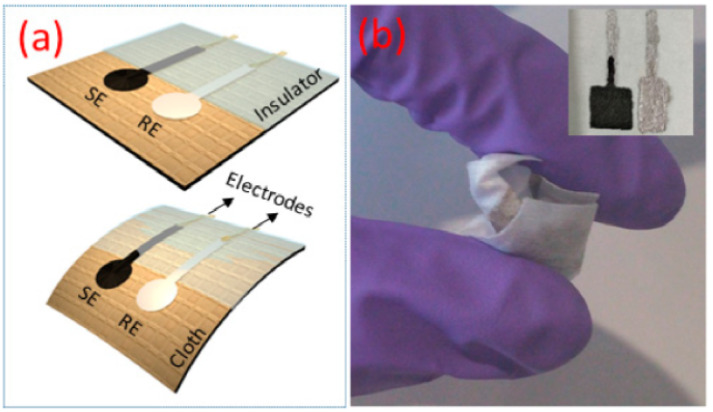
(**a**) Schematic representation of flexible potentiometric pH sensor (SE-sensitive electrode and RE-reference electrode) on cloth. (**b**) The image of flexible and crumpled pH sensor (inset shows the image of the electrodes) [49].

Accurate measurement of soil pH typically necessitates the offline analysis of samples in a laboratory setting. While various on-site measurement methods exist, they often suffer from variability and noise [43,44]. The absence of reliable direct measurement techniques presents an opportunity for developing an on-site system, such as a robotic setup capable of collecting and analyzing samples in real-time. This approach would mitigate alterations in the samples that might occur during transport and address inconsistencies arising from the ambient environment where measurements are conducted. Soil pH significantly influences soil characteristics, especially in forest soils [45]. It affects aspects such as nitrogenous compounds and the soil’s buffering capacity [46]. Overall, pH levels play a crucial role in determining nutrient availability for plants. For instance, in agriculture, phosphorus, a vital plant nutrient, is influenced by soil pH [47]. The linked measurement of pH, while other measurements are taken, allow to generate more environmental data from a larger sample size. Similar to the temperature probes, advances in miniaturizing and making sensors more flexible allow for highly versatile pH sensors that can be easily deployed in a forest environment [48]. Figure 3 represents one such flexible and robust pH sensors.

### 3.4. Imaging Technologies

In the following subsections, we explore a range of advanced imaging technologies, each offering unique capabilities in capturing and interpreting the world around us. We begin with RGB cameras, which provide high-resolution, color-accurate imaging across a wide spectrum. The discussion then shifts to thermal imaging cameras, which detect and visualize infrared radiation. Following this, we examine LiDAR technology, which is a method that utilizes laser light to create detailed three-dimensional representations of environments. The chapter also delves into spectrometers, which are sophisticated instruments that analyze the spectral composition of light. Concluding this section, we discuss multispectral cameras, which capture image data across multiple wavelengths, extending beyond the visible spectrum. These technologies collectively represent the forefront of imaging capabilities, each with its unique method of capturing and interpreting data.

#### 3.4.1. RGB Camera

**Figure 4:**
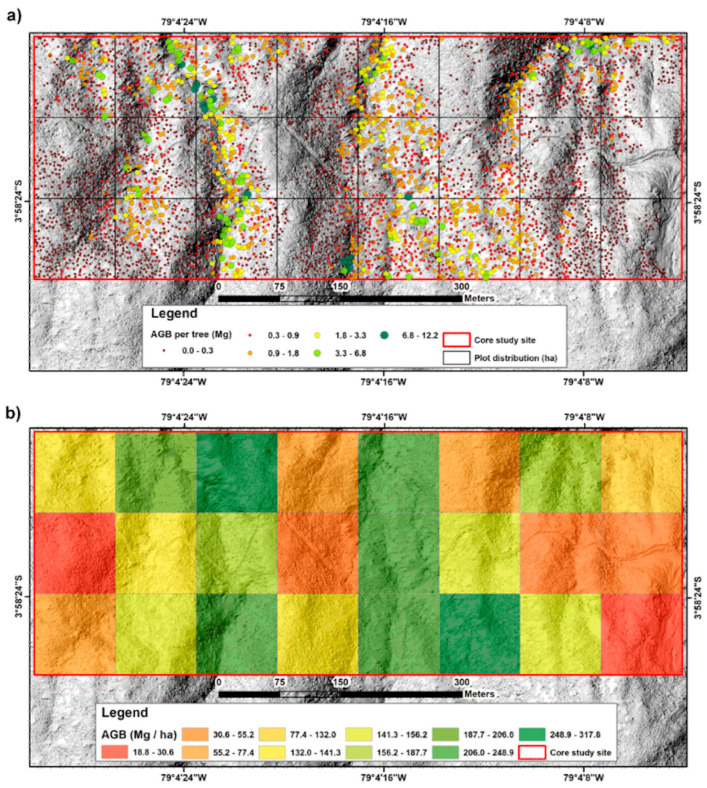
(**a**) Individual AGB values of the dominant trees detected in the core area; (**b**) Spatial distribution of AGB obtained from the RGB data [51].

Advancements in CCD, charge coupled device technology, enabled cameras to become highly efficient and at the same time cheap to operate. These two factors make cameras a versatile input device for digital transformation in forestry. The applications of cameras start with camera traps to monitor wildlife [50] and then range to above ground biomass estimations [51] (Figure 4) and vegetation cover estimation [52]. Cameras additionally are a viable option for the navigation of robots and other autonomous devices [53], which is of high importance to bringing digital transformation into forestry. Most importantly though, cameras are used in a wide array of applications that can have future overlay into the forest industry. An example would be the use of RGB cameras to help vision impaired people navigate in their daily lives [54]. If this technology can be advanced further there is a possibility to use it in challenging terrain navigation.

Cameras pose several failure points that are of high importance in fast moving objects. For pure data generation, one of the most important points to consider is the degradation of the optical equipment. External problems such as dirt on lenses can be overcome relatively easily, but internal damages, for example in the image stabilization, can lead to poor data quality and is complicated to fix [55].

#### 3.4.2. Thermal Imaging Camera

Thermal imaging is a simple technology that can be utilized in a broad field of applications for the forest industry. It ranges from space-based imaging technologies that can be utilized to understand the temperature buffer capabilities of the forest [56], all the way to forest fire detection, where the thermal imaging can be a fast way to locate and observe fire events [57]. These two usecases have a relatively straightforward use of thermal imaging, but it can even be used in the phenotyping of forests [58].

**Figure 5:**
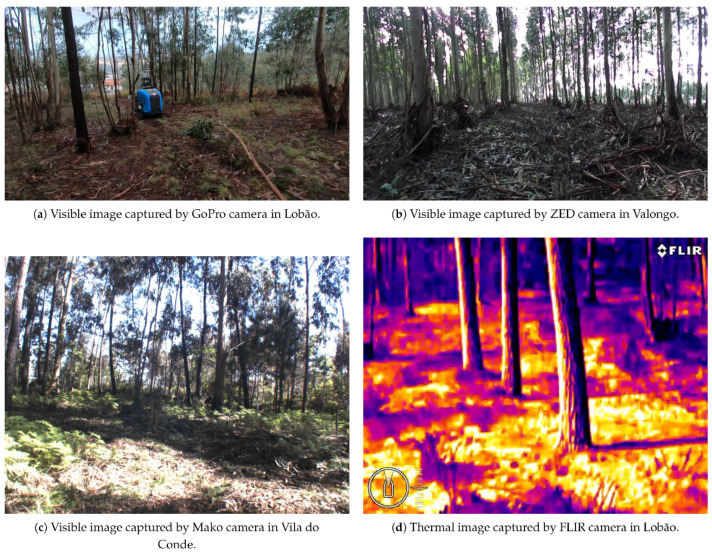
Illustrative images captured by the four cameras in different locations: (**a**) GoPro, (**b**) ZED, (**c**) Mako, and (**d**) FLIR [59].

The recent advances in robots used in forestry led to interesting results on what can be achieved with a relatively basic thermal image. An example of such work was the detection of tree trunks by using a FLIR thermal camera [59] (see Figure 5). The utilization of thermal imaging in forest navigation and data collection signifies a promising advancement. It offers an alternative method for machine guidance in forested terrains, simultaneously gathering essential data. Notably, this approach is implemented through a device that is both straightforward and cost-efficient.

#### 3.4.3. LiDAR

Light Detection and Ranging, or short LiDAR, improved greatly in recent years. Not only did top of the line devices achieve outstanding performance, but more importantly, cheap base model solutions reached performances that could allow the wide deployment of such technologies in the near future. In general, a LiDAR sensor utilizes the reflection of a laser beam to determine a distance of an object by measuring the time it takes a laser to reflect back to its source. This can be conducted very simplified with a unidirectional laser or with a wide array that scans entire three dimensional objects.

**Figure 6:**
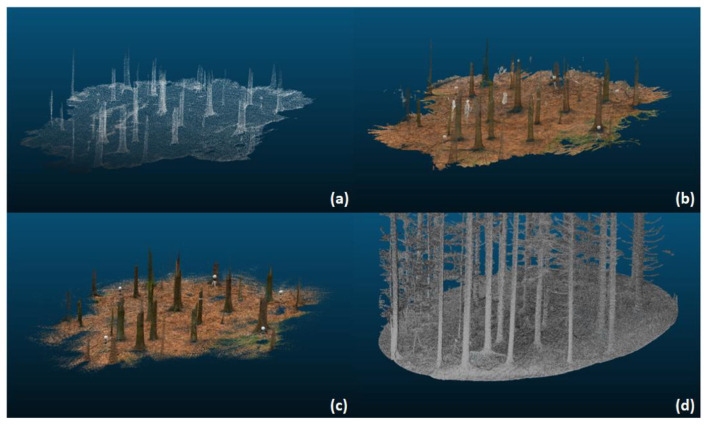
Different forms of digital twins, captured with different technologies (own graphic). (**a**) Point cloud with 3D Scanner App. (**b**) Point cloud with Polycam. (**c**) Point cloud with SiteScape. (**d**) Point cloud with PLS.

This more complex mode of operation allows the construction of point cloud images. Point cloud, because the scanner generates a number of measuring points. These point clouds can be transformed into a so-called digital twin. A digital model of a real space. This can start in digitizing cities [60] and go all the way to digital twins of forests [61], see Figure 6. Such a digital twin gives precise forest inventory data and the described advances in low-cost LiDAR technologies could show their performance in work from 2021 where the built-in LiDAR in an Apple IPad Pro was used to collect the data for digital twin generation [62]. Such advances can greatly help incorporate this technology on a larger scale as pricing is less of a barrier to overcome.

**Figure 7:**
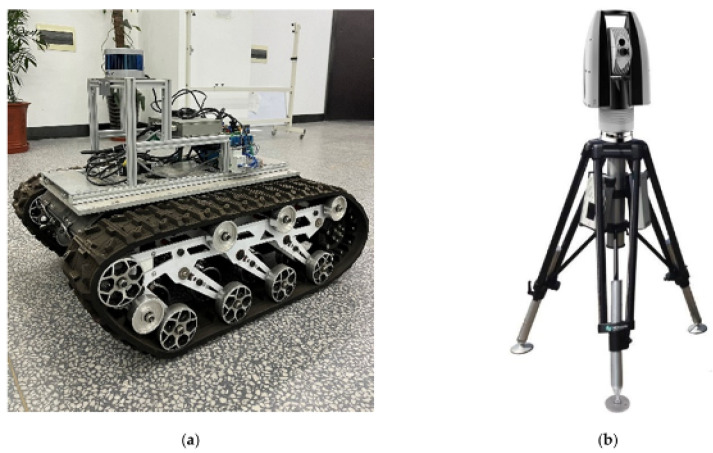
Experimental equipment. (**a**) shows the mobile robot platform and (**b**) shows the Leica Geosystems’ absolute tracker (AT960) [65].

A major downside of LiDAR-derived data is the time needed to collect the data, especially in a forest environment where actual human operators have to carry the device through the forest. There are advances in so-called Personal Laser Scanning (PLS), where the scanner can be moved constantly while taking measurements, as compared to the stationary TLS (Terrestrial Laser Scanning) methods that require a rigid measuring setup that has to be moved between scans [63]. Nevertheless, even with those advances, it is impossible to digitize large forest areas by this method without the expense of massive amounts of human labor. Figure 7 showcases the use of a mobile LiDAR setup compared to a stationary solution; the mobile platform can be a major turning point in allowing a larger amount of data to be collected without the expense of large amounts of human labor. This makes the data collection decoupled from the human operator and fully autonomous. Another promising option for terrain that can be observed from above is the use of aerial LiDAR. In this case, the scanner becomes attached to a drone and is flown over the area of interest. This can drastically speed up data collection, showing comparable results, for example, in the structural diversity of forests in the US [64]. The important factor here will be the combination of both technologies, as above and below tree canopy data can be useful when an aerial image alone is not able to capture all data required. One major benefit is the possibility to cover large areas fast and therefore have the ability to take repeated measurements, allowing almost real-time data updates for certain forest areas.

An important factor that can be limiting in the use of LiDAR is precipitation, especially when the data should be generated from a larger observed area. With an increase in rainfall, the detectable distance drastically decreases [66]. For pure navigational purposes in close proximity to the scanner, heavy rain is required to completely nullify the data generated, but with the overall goal of high-quality data generation for digital transformation, rain should be avoided wherever possible.

#### 3.4.4. Spectrometers and Multispectral Cameras

**Figure 8:**
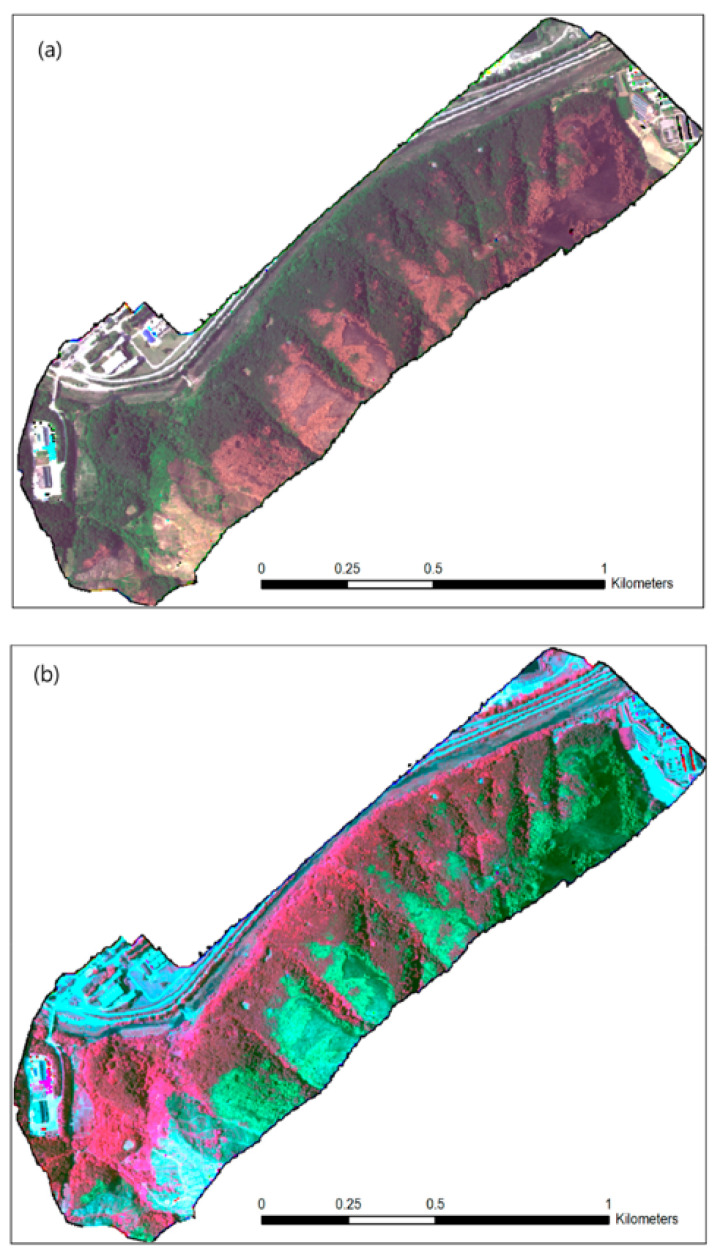
RedEdge multispectral unmanned aerial vehicle (UAV) image of some of the burned area near the city of Gangneung: (**a**) natural color composite: RGB = band 3, 2, 1; and (**b**) pseudo-infrared composite: RGB = band 5, 3, 2 [71].

Generally speaking, spectrometers are used to detect various wavelengths of light, and a multispectral camera has the ability to capture a broad spectrum of light. This is important, as this technology is not limited to the visible range of light. The spectrum can extend to infrared in the longer wavelengths and to ultraviolet in the lower ones, practically allowing one to observe an object more completely than possible with the human eye alone. Such imaging techniques are useful tools for the determination of tree species and the vitality of trees. A prominent example is bark beetle infestations, where multispectral imaging can be used to detect unhealthy specimens within a forest [67]. This technology can not only be used to determine the health status of trees, but it is also possible to distinguish between different species. The identification of invasive species on a larger scale [68] is an example. An example of a multispectral image can be seen in Figure 8.

The advantage of multispectral imaging comes in its relatively simple setup that can be easily customized and utilized in various applications. An example is presented by Montes de Oca et al., where a low-cost setup for crop monitoring [69] is developed. The relative ease of the setup allows for a wide application in different fields and can be an add on to existing data collection systems.

Spectrometers also have additional benefits, for example, robot olfaction, utilizing Tunable Diode Laser Absorption Spectroscopy (TDLAS) [70]. Here, the possibility arises to detect gases remotely, be it for the assumption of greenhouse gas levels in a certain area or to detect potential explosion hazards. Greenhouse gas measurements could be a great add on to an already moving system in the forest.

### 3.5. Observation Platforms

#### 3.5.1. Satellites

The application of satellites for remote sensing is a practice with a history spanning several decades [72,73]. Annually, an increasing number of satellites, equipped with progressively advanced sensors, are launched into orbit. A notable example of freely accessible satellite data is provided by the Copernicus Earth observation program. The satellites employed in this program encompass a diverse range of data types, from thermal imaging and radar imagery to intricate spectral images, that offer insights into the chemical composition of the atmosphere.

The large advantage of satellites for sensor applications comes from the fact that huge areas can be monitored without the need to physically gather the data. This ability allows for the long-term monitoring of areas in a very cost-effective way. An assortment of satellite data can be used to evaluate forest health [74], above ground biomass [75], or as an addition to real-time forest inventory data [76].

The major downside of satellite-obtained data lies in the fact that the data collection is far away from the data you want to obtain; this is obvious in the case of data that is related to the ground level like forestry. When it comes to imaging, weather is an important factor that can, for example, hinder the use of optical sensors. A thick cloud cover can completely stop the data obtainable in a certain period of time.

Conversely, it is crucial to acknowledge the limitations of satellite-based data acquisition, particularly its inability to capture information beneath the tree canopy. This inherent constraint underscores the importance of integrating satellite data with other data sources for a more comprehensive understanding. This concept of data integration is not new; a decade ago, researchers recognized the value of combining aerial data with ground-based observations, as exemplified in the study by Pratihast et al. (2014) [77]. This approach enhances the depth and accuracy of an environmental analysis by merging the broad, top-down perspective of satellite imagery with the detailed, localized insights gained from ground-level data.

**Figure 9:**
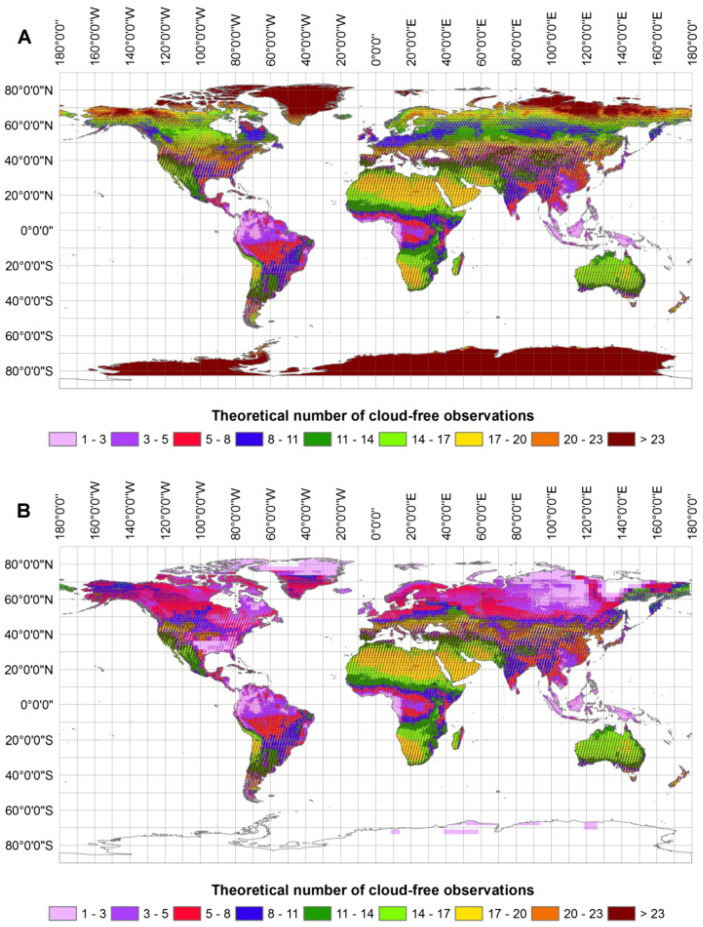
Obtained from Wulder et al. [78]. Approximate number of cloud-free LANDSAT observations per year (inset (**A**)), assuming the 2013 mean annual cloud fraction, as estimated from MOD35 [83,84] and the WRS-2 Path Rows with a 16-day revisit cycle (descending nodes only). Approximate number of cloud-free LANDSAT observations within the growing season (inset (**B**)). The actual number of useful observations may be further limited by the length of the growing season and availability of daylight, particularly in higher latitudes. Growing season was approximated as number of days with a mean daily air temperature >+5 °C.

Another significant development lies in the use of satellite constellations, combining multiple satellites with similar sensory equipment to obtain better coverage of a certain area of interest. Not only in terms of sensory equipment but also general coverage that can be obscured by weather, as mentioned above [78], see Figure 9.

Satellite imagery, while useful at a regional level, often lacks the detailed information necessary for effective decision-making in applied forestry [79]. Applications for local forest inventory, planning, or damage monitoring have observed limited success and reliability. While satellite images can be effective for stratification in multi-stage sampling and monitoring clear-cuts, the associated costs may not always be justifiable. Many studies have oversimplified the information needs in forestry planning, assuming that basic forest mapping holds significant value without directly linking it to management decisions. Despite advancements in complex reflectance modeling, considering factors like internal shading and topography, these developments have not markedly enhanced outcomes compared to earlier efforts.

With satellite remote sensing and digital image analysis no longer being technologically groundbreaking, it may be time to acknowledge that current satellite sensors generally fall short for forestry planning due to their limited relevant information and the existence of more efficient data collection methods for forest management planning [80,81,82], as we will now discuss in the further course of this paper. It is important to note that satellite-based systems undergo continuous improvement, but for future AI applications, more than aerial data will be required for the optimal performance of such a system. In their current state, the data will be utilized but cannot be regarded as the one and only source for forest management.

#### 3.5.2. Unmanned Aerial Vehicles (UAVs)—Drones

Unmanned Aerial Vehicles (UAVs), commonly known as drones, have become a cornerstone in the realm of remote sensing generally, necessitating their mention alongside traditional remote sensing tools. While UAVs themselves are not sensors, they serve as platforms for carrying various sensors, thereby playing a central role in data acquisition. The emergence of drones has revolutionized remote sensing primarily due to their cost-effectiveness compared to traditional manned aircraft such as planes and helicopters. This economic advantage has made UAVs accessible for a wide array of applications, ranging from academic research to commercial use.

Moreover, drones offer flexibility and accessibility and are capable of operating in diverse environments and reaching areas that are otherwise challenging or risky for manned aircraft. This capability significantly expands the scope of remote sensing, encompassing difficult terrains and sensitive ecological zones. One of the most notable advantages of UAVs is their ability to capture high-resolution imagery by flying at lower altitudes than manned aircraft. This feature is crucial for detailed analysis and monitoring in various fields, including smart (precision) forestry. A good example of the capabilities of drones in Smart Forestry is species identification from aerial images obtained by drones [85]. In agriculture, drones can be used to access crop health, obtain 3D terrain maps, or estimate soil moisture, and, most importantly, all of this must be conducted without any disturbance to the crop itself [86]. In an urban setting, drones offer the same benefits of simple and easy deployment for different parameters; one example, in this case, is the surface temperature measurement in urban settings used to differentiate between different surface types and their impact on temperature [87].

**Figure 10:**
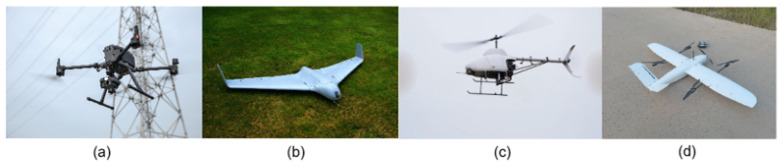
Different UAV platforms: (**a**) Multi-rotor UAV, (**b**) fixed-wing UAV, (**c**) unmanned Helicopter, and (**d**) VTOL UAV, obtained from Zhang et al. [90].

In the context of smart forestry, drones have been a method in practice for several years, with a notable surge in popularity over the past decade [88], which is especially due to the improved load capacity and flight characteristics. This increase can also be attributed to the declining costs of drones and their accessories, making them a more accessible tool for various forestry applications. The integration of AI promises to further refine these applications [89]. One of the primary applications is in tree species mapping and classification. Drones equipped with advanced sensors can capture high-resolution images, enabling the identification and classification of different tree species, which is an essential aspect of forest management and biodiversity conservation [82]. Another significant application is in forest fire detection and damage monitoring. UAVs can rapidly survey large forest areas, providing real-time data that are crucial for the early detection of forest fires. This capability not only aids in prompt firefighting efforts but also helps in assessing the extent of the damage post-fire, which is vital for recovery and reforestation planning. Additionally, drones are used for monitoring forest health and growth, detecting illegal logging activities, and aiding in wildlife conservation efforts. They offer a bird’s-eye view that is particularly useful for the large-scale monitoring and management of forest resources. The flexibility to equip drones with various sensors, such as multispectral and thermal sensors, further enhances their utility in forestry. Figure 10 indicates the diversity of UAVs employed in this sector. Different drone models and configurations are selected based on specific requirements such as flight duration, payload capacity, and the type of sensors needed for particular forestry operations.

The link between the current state of the art in drones and AI comes in the form of utilizing regular flights that gather different data points. This regular recording of the same area provides more detailed information that comes closer to real-time monitoring. Additionally, having multiple inputs from each flight AI can not only utilize the data for one specific application. Thermal images, for example, could be checked automatically for high temperature spots that could indicate a beginning forest fire. At this point, the human-in-the-loop approach comes into play again. If an AI detects an anomaly it cannot categorize, a human expert will be used to resolve the issue. For example, the high-temperature spot detected could be a hot exhaust pipe of a forest machine, and a human can detect something like this easily by comparing images. On the other hand, it could be that there is a possibility that it is a fire, and measures can be taken to check the location or directly set actions for fire fighting.

## 4. Sensor Network Operation

Sensors have been employed across various industries for many years. A significant challenge in forestry is the deployment and operation of multiple sensors within forest environments; for reaching a certain autonomy, the integration of multimodal sensor systems in forestry machines is necessary [91]. Establishing effective communication among sensors is a primary concern. Wireless technology, commonly used for this purpose, has been extensively studied, even though sensors may produce different types of data. Lewis (2004) [92] has thoroughly investigated the complexities associated with sensor communication and wireless connectivity. In forest settings, additional challenges arise due to signal instability, necessitating the implementation of multiple transmission methods. One such method is Low-Power Wide-Area Network communication, which facilitates device communication in remote areas with weak signals, as explored by Zhao (2023) [93]. A critical aspect of this approach is ensuring that the entire network connects to a location with reliable internet access for data transmission. However, a limitation of this technology is the need for a fully connected network, which, while feasible on a smaller scale, poses difficulties in monitoring large forested areas. Operating a robot network is one of the most promising technologies for forestry in the future. It is important to know the correct specification of existing robots for use in the right place or environment [94]. As outlined in the sections above, it can be a major advantage to link devices that can help a robot maneuver the environment with data that are valuable to forest operators and researchers. A recent study evaluated how different perception technologies are used in conjunction with robots today. A major downside identified in this study was the non-existence of robot teams within forestry [95].

**Figure 11:**
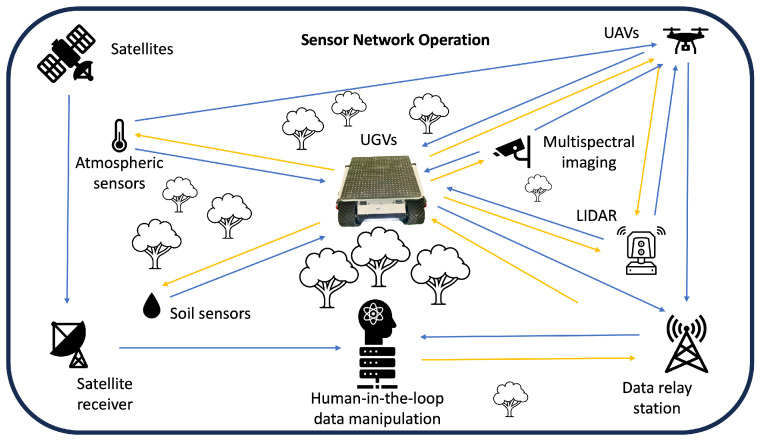
Our sensor network framework for smart forestry with a robotic base that interacts with various sensors. Blue arrows represent data transfer; yellow arrows show direct interaction, e.g., maintenance, setup, etc.

The use of a fully mobile setup with a main “hub” that can collect and manipulate data while moving through the forest can bring the benefit of using sensor networks, by grasping their data every time it passes by without the need for an extensive network throughout the entire area. Another option is the combination of multiple robotic systems into one team that can cover the challenges in forestry. An example for such a system was proposed and tested for data collection at construction sites. In this case, a wheeled robot was paired with a blimp to have ground and aerial coverage of the area of interest [96]. Developments of such systems in forestry can greatly enhance the data collection capabilities with all kinds of stationary or mobile sensor setups. Our vision of a forest sensor network is depicted in Figure 11.

Expanding upon the concept of collaborative operations in challenging forest environments, the integration of robots, drones, and human expertise forms a comprehensive framework for teaming-up. Recent advancements in swarm robotics have significantly enhanced their problem-solving abilities, as evidenced by the work of Garattoni (2018) [97]. The deployment of a mobile ‘hub’ system, capable of traversing the forest and collecting data from sensor networks as it passes, offers a solution that negates the need for extensive fixed networks across the entire area. This hub can act as a central point for data aggregation and manipulation, harnessing the information gathered by stationary or mobile sensors.

Another approach involves the synergy of multiple robotic systems, creating a team that addresses the multifaceted challenges of forestry. A pertinent example of this concept in action is observed in the combination of a wheeled robot with a blimp for data collection at construction sites, as explored by Asadi (2020) [96]. This ground and aerial team provides a comprehensive coverage of the targeted area. Applying similar collaborative systems in forestry could significantly enhance the data collection capabilities, utilizing an array of stationary and mobile sensors.

Our vision for a forest sensor network, incorporating these elements of robotics, drone technology, and human-in-the-loop, is illustrated in Figure 11. This integrated approach promises to revolutionize the data collection and monitoring in forest environments, leveraging the strengths of each component to create a more efficient, effective, and comprehensive system.

The most critical challenge for future smart forest operations lies in the off-grid energy supply for sensors. While sensors mounted on robotic platforms derive their energy from the platform itself, the powering of these platforms is beyond the scope of this paper. The focus is on small, remotely located sensors that require autonomous power sources. Presently, solar power is the primary tested solution for such applications. However, the effectiveness of a photovoltaic system hinges on the placement of the solar cell, which must receive sufficient sunlight to maintain a battery charge for continuous operation. Addressing this limitation involves reducing the overall power consumption of the sensor setups and enhancing the efficiency of the power input, as discussed by Boehm (2023) [98]. Furthermore, to ensure a sustained operation, the implementation of autonomous maintenance, such as battery replacement, potentially carried out by an autonomous robot, is a vital additional consideration.

## 5. Robot Integration

The construction of a robust and capable robotic base is a key cornerstone to achieving widespread data generation in forestry with existing technologies. This robotic base needs to fulfill multiple tasks at once.

First of all, the unmanned operation needs to be achieved, transforming the simple robot into an unmanned ground vehicle (UGV) or autonomous mobile robot (AMR). This task requires the robot to navigate the environment autonomously. To achieve this, inputs from various sensors are required, especially in challenging terrain like forests [99]. Robot navigation is one of the major challenges where the human-in-the-loop approach can be of great benefit. A good example is a robot that encounters an obstacle that cannot be overcome by the robot alone. In such a case, there can be a quick connection established with a human operator that can most of the time quickly resolve such an issue just by camera images and allow the robot to continue its operation. A concrete problem would be the detection of high grass compared to a wall. Today, most robots cannot handle high grass, but a human can resolve this issue within seconds.

With this, we want to outline that the robot will need a human to interact with to overcome certain challenges, and it is unavoidable that sometimes actual physical human interaction is the only way to overcome these challenges. An autonomous setup is tailored to minimize these instances and allow the robot as much as possible.

Second, enough energy needs to be available to power the robot, sensors, and the entire network in an unstructured off-grid environment. Power provisioning is key to enable a reasonable operational time for such systems without the interaction of human operators. Recent developments explore a concept called embodied energy, trying to avoid separate power provisioning systems like batteries by directly incorporating them into the robot’s structure [100].

From looking at the order of things required to enable an autonomous system, our focus was clearly focused on the construction of a simple yet robust setup to enable the use of all kinds of sensors to operate the robot and generate the data required for digital transformation in forestry. Therefore, the next section outlines the first step we took toward an autonomous data generation system: a standardized equipment carrier.

### Equipment Carrier

**Figure 12:**
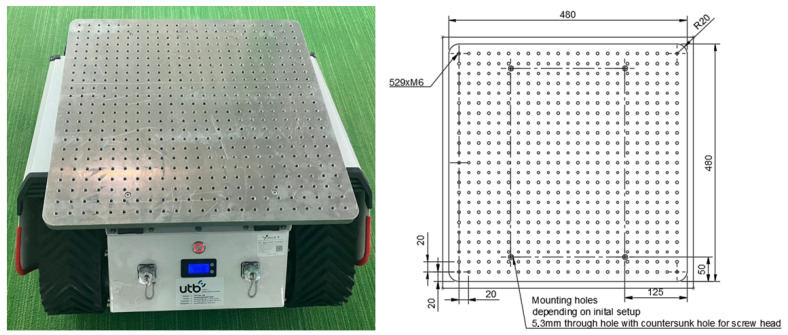
Our universal sensor-carrying platform mounted on AgileX bunker mini (AgileX Robotics, Shenzhen, China) and the corresponding technical drawing.

The integration of a UGV as a centerpiece for data generation requires the possibility of operating multiple sensors from this platform. A standardized equipment carrier, consisting of a 15 mm aluminum plate prepared with a regular grid of 20 mm spaced M6 threads to enable the easy and perfect orientation of different equipment every time, was developed as a starting point. Three-dimensional-printed adaptors are used for equipment with different mounting spacing. In addition, the ability to always have the exact same location of a sensor on the robot allows for fast spatial relating between different sensors. E.g., two GNSS dishes mounted on opposite sites of the platform to provide bearing data do not need to be measured to know their exact position in relation to one another, and the carrier itself is given by the plate. Every piece of equipment can be mounted on the plate with standard M6 round head screws, and 3D printing allows for a rapid adjustment for devices that do not follow the regular grid pattern. The plate itself can be moved to different robotic systems, making it a flexible testing platform that can bring rapid performance data from different chassis options, while at the same time keeping variability to a minimum. A technical drawing of the platform can be observed in Figure 12 together with a tracked robot as a chassis option.

This mounting plate is able to not only accommodate sensors but additionally carry robotic arms/manipulators that can interact with the environment. In particular, looking into cheap options based on the already mentioned Arduino or Raspberry Pi systems offers possibilities that were not available before [101]. This advancement facilitates indirect measurements, such as soil pH, and is crucial for the deployment and maintenance of sensors. Our system can be compared to space exploration technologies such as the Mars Rovers, which collect and analyze samples [102], reflecting the advancements we have collectively achieved in our terrestrial system.

## 6. Improving Databases

There are several open databases in the fields of smart forestry and climate research that are maintained by the international community. These databases allow for the storage and sharing of sensor data and other research findings within the international research community. Some notable examples include:

- ForestPlots.net: A global network that brings together researchers and forest plots data to monitor the dynamics of tree communities in response to environmental change [103].
- Global Forest Watch (GFW): An interactive online forest monitoring and alert system designed to empower people everywhere to better manage forests. It uses satellite imagery and other technologies to provide data about forests worldwide [104].
- FLUXNET: A global network of micrometeorological tower sites that use eddy covariance methods to measure the exchanges of carbon dioxide, water vapor, and energy between the biosphere and atmosphere [105].
- OpenTopography: A portal that provides access to high-resolution, Earth science-oriented, topography data, and tools, including data from LiDAR surveys [106].
- Earth System Grid Federation (ESGF): A web-based tool hosting a multitude of datasets related to climate science, including outputs from various climate models and observational data [107].
- TerraClimate: A dataset of monthly climate and climatic water balance for global terrestrial surfaces, providing high-spatial resolution data.
- NEON (National Ecological Observatory Network): Provides open access ecological data, including sensor data, from across the United States [108].
- TerraClimate: A dataset of monthly climate and climatic water balance for global terrestrial surfaces, providing high-spatial resolution data [109].

A huge benefit from automated data collection can come in the form of improved databases. Robotic systems can be programmed in such a way so as to have a high repeatability in sample measurement. This high repeatability can be the stepping stone into a broad range of databases featuring different kinds of environmental data. Linking this to the previously mentioned data requirements of AI to function properly, there is huge potential in a broad environmental database.

The big picture after implementing a broad range of sensing methods for an autonomous system would be the generation of data in a specific area but linking all of these data together. The exact GPS position is linked to an exact digital twin of the surroundings with weather data from that exact location linked to soil and atmospheric parameters. Such databases could open up completely novel discoveries by revealing a connection between parameters that might even stretch across multiple fields of research and therefore are so far undetected.

A prominent example could be a solution for the detection and prediction of bark beetle calamities. As of today, there is no way to predict bark beetle outbursts, but there might be an underlying connection that could not be detected so far, and the use of AI linked to high quality multi-field data might solve this problem.

## 7. Challenges and Future Work

This paper explored some of the most common sensor technologies that can be utilized in forestry. This is a good starting point for the much needed data generation. Nevertheless, there are still challenges that need to be tackled.

First and foremost, enough sensors need to be deployed, and their data need to be available in one location to benefit from AI. Existing data can be used but often lack the connection between data, meaning they do not have the same location and time of acquisition. Both factors are critical to help an AI establish a meaningful connection between different sets of data. Similar to what a weather station does today, all parameters are taken into account from the same location at the same time.

To enable this widespread generation of data, the automated collection is key. Complex setups like imaging technologies only add value if enough area can be covered and digitized in this way. A simple system is required that automates the data generation. As mentioned above, we envision a robotic backbone system to achieve this goal, but there are other opportunities that can be explored. For example, affordable and standardized LiDAR scanners could be mounted on existing forest equipment to generate larger amounts of data with equipment that is already present in the forest today. Movements in this direction could greatly enhance the digital transformation of the forest industry.

Last but not least, all of those systems need to be able to be used on a large scale by the industry and not only research, which additionally puts great emphasis on the economic feasibility of such solutions. Capital as well as operational costs need to be considered in the development to ensure that the added value of digital transformation can be achieved not only in research but, more importantly, in the industry itself.

Our goal for the future is the set up of an autonomous robotic data collection system that can operate the vast forest road networks on its own and start generating data. The expansion will be the integration of different technologies that allow the system to venture out into the entire forest terrain. As those are very ambitious goals, we want to start by setting up the baseline system for the robotic data generation platform and low-cost sensor setups that can be integrated into equipment that is already moving through forests on a regular basis.

The direct usability for different forest experts can firstly be obtained by feeding databases with high-quality data frequently. Having access to comparable data from various locations can aid the work of individual scientists by giving them access to more data than they would be able to obtain from their experiments. There is a possibility to check whether certain models that work for a certain area can be utilized for different ones by checking results with data that are already available in a database. We want to emphasize that these databases will be simple in the beginning but the power behind the automated data collection is the ability to grow them exponentially over time. As soon as these data are available, models can be set up for any research question that relies on data that can be obtained in the way outlined in this manuscript. This is where AI with the human-in-the-loop approach will become an indispensable tool for the forest expert. The experts will have the ability to aid in answering their given research question by seeing new connections between types of data that have not been known or used so far. The ability to compute highly complex connections between data types and then feed them to the expert, and also obtain additional input from the expert, makes AI in forestry an excellent tool with difficult and multidimensional questions.

## 8. Conclusions

The selection of sensors suitable for integration into a sensor network highlighted that the most effective configurations often involve sensors connected to an autonomous mobile data collection device—a robot device. Contemporary sensors are versatile, catering to a wide array of applications ranging from forest fire detection to forest inventory. These sensors have undergone extensive testing and applications in various industries outside of forestry. The future challenge for the research community lies in adapting and implementing these proven sensor technologies within practical forestry contexts. This adaptation is crucial for addressing real-world forestry issues and leverages the established reliability and versatility of these sensors to enhance forest management and conservation efforts. Artificial intelligence stands as a pivotal force in the ongoing digital transformation, offering novel solutions across various domains. However, it is essential to recognize that the foundation of AI’s success lies in the quality and quantity of data it utilizes. This underscores the critical role of sensors as the primary source of data; even the most advanced algorithms are reliant on the data provided by these sensors. Prioritizing the enhancement of data quality through an improved sensor technology is, therefore, more consequential than refining the algorithms themselves. High-quality sensors yield more accurate and reliable data, which are fundamental for effective AI applications. Despite their importance, modern sensors also present significant challenges and untapped potential. They may introduce new, unforeseen risks, necessitating a heightened focus on security, traceability, transparency, explainability, validity, and verifiability [110,111]. As sensor technology continues to evolve, ensuring these aspects becomes increasingly vital to safeguard against potential threats and to maintain the integrity and reliability of the data they collect. This comprehensive approach to sensor development and data management will be crucial in maximizing the benefits of AI in the digital transformation era while mitigating risks and maintaining trust in these technologies.

The implementation can reach from pure stationary sensors connected via an internal forest network to autonomous robots moving through an area connecting not only stationary setups but also providing data themselves. The utilization of technologies already used in different industries today can be a stepping stone to bringing more elaborate sensor setups into forestry. We envision a future of robot teams maneuvering through the forest autonomously, collecting data while navigating. Ground-based robots can have the ability to place and maintain stationary sensors and be a base for other robots, like UAVs, that collect additional remote sensing data when required. A connection with existing infrastructure like satellites shall be established, enabling the entire repertoire of information available to be used and processed together The use of a standardized system for data collection will play a major role in allowing machine learning to excel in the forest industry, by providing optimal data to the algorithms. Especially when the human-in-the-loop approach is practiced, it will be possible to obtain the best of the automated data collection and workload reduction, while still having the expert knowledge and intuition of human operators in solving problems.

Finally, the utilization of data generation on a larger scale opens up possibilities currently unavailable. Having the opportunity to gather data, not only from certain predefined areas but most parts of the forest environment, opens up solutions for collaborative research and data connection that could not be identified up to this point and might arise due to a broader understanding of forest parameters.

## Figures and Tables

**Table 1:** Sensors in Smart Forestry.

Sensor Type	Application	Advantages	Disadvantage
GNSS and Wifi, etc.	Localization	Accurate position determination	Problems in covered terrain due to signal inconsistency
Temperature sensors	Temperature monitoring	Provide insight into temperature fluctuations and can be linked to multiple other inputs (e.g., moisture), available to measure temperature in different media	Without linkage to other data, has a very narrow application in forestry. Continuous monitoring requires a good network
Moisture and humidity sensors	Measuring water content in air or soil	Produces climate data and can be easily linked to inputs like temperature. Applications can range from humidity measurements under the canopy to determining the trafficability of forest roads.	Real-time monitoring is only possible with a good network. Setup can be complicated.
Soil pH	Determining the solubility of different nutrients in soil	Understanding the nutrient uptake capability in relation to the soil.	On-site measurements with embedded sensors cannot reach the accuracy as an in-lab measurement
RGB camera	Generates images of the surroundings	Wildlife monitoring, understanding forest road condition, and navigational aid	Processing of pure image data can be challenging. Large amounts of data are generated when high-quality video/photo is used
Thermal imaging camera	Measures heat profiles of the surroundings	Understanding of thermal gradients; useful in phenotyping; forest fire detection SAR	Large amount of data generation possible, and image processing can be challenging.
LIDAR sensor	Provides detailed topographical data	Assists in biomass estimation, forest structure analysis, and navigation	Most commonly used technologies today are expensive. Problem with rain or other contamination on the transparent window.
Multispectral cameras	Measuring multiple spectra at the same time for simple data linking	Simple setup to obtain a broad range of data that is directly linked to one another	The collection of multiple wavelengths at the same time can lead to poor performance of each detector compared to specialized ones

**Table 2:** Sensor Operation Platforms.

Device	Application	Advantages	Disadvantage
Satellites	Remotely detect various data inputs (Temperature, moisture, images, etc.)	Ease of access to data, standardized datasets, and ability to monitor almost the entire surface of the earth	In many cases dependent on weather conditions. Continuous monitoring of a certain area is hardly possible. Large distance to the area of interest.
Drones	Remotely detecting various data inputs.	Cheap and easy to operate by a human; operation below the tree canopy is possible	Limited operation time for non-fixed wing variants; challenging to autonomize for flight below the canopy
Unmanned ground vehicles	Autonomous data collection and interaction with ground-based equipment. The potential communication link with other equipment like drones	Longer operation time compared to drones, with a high payload and is simple to use	Without external computing sources, fully autonomous maneuvering is still not possible
Existing forest machinery	Data collection	Simple sensors that do not require a lot of human interaction can gather data without interfering with the human workflow.	Complex sensors pose issues due to harsh operating conditions, e.g., LIDAR on a harvester. Therefore, they are limited to simpler sensors

## Data Availability

No new data were created or analyzed in this study. Data sharing is not applicable to this article.

## Abbreviations

The following abbreviations are used in this manuscript:

AI	Artificial Intelligence
AGB	Above Ground Biomass
AMR	Autonomous Mobile Robot
ESGF	Earth System Grid Federation
FLIR	Forward Looking InfraRed
GFW	Global Forest Watch
GNSS	Global Navigation Satellite System
GPS	Global Positioning System
LiDAR	Light Detection and Ranging
MFC	Microbial Fuel Cell
ML	Machine Learning
NEON	National Ecological Observatory Network
PLS	Personal Laser Scanning
PV	Photovoltaic
RFID	Radio Frequency Identification
RGB	Red–Green–Blue
TDLAS	Tunable Diode Laser Absorption Spectroscopy
TLS	Terrestrial Laser Scanning
UAV	Unmanned Aerial Vehicle
UGV	Unmanned Ground Vehicle
VTOL	Vertical Takeoff and Landing
